# Correction: Developing a patient flow visualization and prediction model using aggregated data for a healthcare network cluster in Southwest Ethiopia

**DOI:** 10.1371/journal.pdig.0000455

**Published:** 2024-02-07

**Authors:** Balew Ayalew Kassie, Asaye Birhanu Senay, Geletaw Sahle Tegenaw

Asaye Birhanu Senay should be included in the author byline instead of the Acknowledgments. Asaye Birhanu Senay should be listed as the second author, and his affiliation is:

3. Faculty of Public Health, Institue of Health, Jimma Univeristy, Jimma, Ethiopia

4. Jimma Univeristy Capacity Building and Mentorship Program, Jimma, Ethiopia

The contributions of this author are as follows: Conceptualization, Data curation, Formal analysis and methodology.

The correct citation is: Kassie BA, Senay AB, Tegenaw GS (2023) Developing a patient flow visualization and prediction model using aggregated data for a healthcare network cluster in Southwest Ethiopia. PLOS Digit Health 2(11): e0000376. https://doi.org/10.1371/journal.pdig.0000376
