# Supplementary material for: Developing a patient flow visualization and prediction model using aggregated data for a healthcare network cluster in Southwest Ethiopia
Source: PLOS Digit Health. 2023 Nov 8;2(11):e0000376. doi: 10.1371/journal.pdig.0000376 (PMC10631638; doi:10.1371/journal.pdig.0000376)
Supplement: Attachment — Submitted filename: Response to Reviewers.docx [file pdig.0000376.s001.docx]

Dear Sir/Madam

Thank you for allowing us the opportunity to submit a revised draft of the manuscript. We thank you and the reviewers for a thorough reading and constructive criticism of our manuscript and for the opportunity to revise and resubmit. We are pleased to submit the improved research article, including a proposed slight title change, “**Developing a Patient Flow Visualization and Prediction Model using Aggregated Data for a Healthcare Network Cluster in Southwest Ethiopia**” for your consideration in the PLOS ONE. On the following pages, you will find our response to the editor and reviewer comments.

On behalf of my co-authors, I thank you for your consideration of this resubmission. We appreciate your time and look forward to your response.

Please see below, in blue, for a point-by-point response to the reviewers. All modifications in the manuscript have been highlighted in red.

Sincerely,

Balew Ayalew Kassie

Reviewer #1: Patient flow in healthcare systems demonstrates patient interaction or movement through various service points (from the point of registration to discharge common for both inpatients and outpatients). Therefore effective health information systems depict various patient interaction points. Identifying challenges at each of these interaction points is a sure way of dealing with patient flow problems such as overcrowding, scheduling, reducing queueing/waiting time, improving quality of service, etc. at a specific service point. (World Health Organization, “Strengthening health information systems,” Strength. Heal. Inf. Syst., pp. 1–4, 2017)

(Q. Nguyen, M. Wybrow, F. Burstein, D. Taylor, and J. Enticott, “Understanding the impacts of health information systems on patient flow management: A systematic review across several decades of research,” PLoS One, vol. 17, no. 9 September, pp. 1–20, 2022, doi: 10.1371/journal.pone.0274493.)

The author's description of patient flow both in the text (abstract section), results, and Figure 1 in this context can be described as a statistical description of hospital attendance and is inconsistent with patient interactions (flow) in any health information system.

Thanks for the comment. We acknowledge and recognize the validity of concerns raised regarding the current body of research. As per our understanding and experience in low-resource settings, the majority of the existing literature focuses on intra-patient flow analysis, which concentrates on the movement of individual patients within a single healthcare institution and between different departments within the institution. Mainly to explore the length of stay, waiting time, treatment time, test turnaround time, and boarding time. However little is known and explored about the flow of patients with the network structure or organization. For example, Ethiopian healthcare is structured in a 3-tier fashion, where the referral patient flow starts from the lower primary healthcare level to the secondary level on the way to the tertiary level or a specific hospital. Therefore, to fill this gap, our study's goal was to investigate the flow of patients in the Healthcare Network Cluster in Southwest Ethiopia. Our inter-patient flow analysis looks at the movement of patients between different healthcare institutions, such as from a health center to a primary hospital to a general hospital on the route to a specialized hospital. We believe inter-patient flow analysis can help identify areas for improvement in the coordination of care and the efficient use of resources by tracking the flow of patients from one primary healthcare provider to specialized care and analyzing the patterns and characteristics of that flow (the volume of the flow at the current research stage). It is vital for successful planning and quality management to analyze the flow of referrals in and out. This process helps healthcare practitioners understand the volume of flows, which can have a significant impact on admissions, bed occupancy, and length of stay. This research might potentially be utilized as a first step to developing methods for analyzing the flow of patients for advanced preparation, planning, and management. Furthermore, the study only takes into account direct flow patients who use or follow the traditional healthcare system structure, not indirect flows or alternative forms of healthcare access. The study's assumptions and findings may not apply to different demographics or healthcare settings.

Reviewer #2: In this research article, the authors implement two machine learning methods to predict the outpatient flow from several health facilities in Ethiopia, and use Sankey diagrams to visualize these flows. The article is clear and easy to follow, and the problem it seeks to address is both important and well-characterized. However, I have two major comments:

a. Details about testing and training datasets (e.g. how were training/test sets divided, was cross-validation employed?)

The dataset was divided into training and testing tests. and one-third of the dataset was used for testing.

b. Model hyperparameters (e.g. how many trees were in the RF, how many neighbors were used in the KNN model, how were hyperparameters optimized?)

We used the default parameters for the random forest decision tree classifier, including the values of n_estimators, min_samples_leaf, and min_samples_split, which are equal to 100, 1, and 2, respectively. In addition, we employed hyperparameter tuning with different values, including n_estimators, max_depth, min_samples_split, and min_samples_leaf. The n_estimators of 50, 100, and 150 were used. The max_depths were None, 10, and 20. Min_samples_split was 2, 5, and 10. The min_samples_leaf was 1, 2, and 4. Whereas the hyperparameter optimization was used in KNN with n_neighbors values of 3, 5, and 7 as well as weights with a value of uniform and distance.

c. How were the thresholds between quiet, optimal, and busy determined, and what is the utility of binning the flows this way instead of using a continuous or binary outcome?

The quiet, optimal, and busy thresholds were identified by analyzing the distribution of patient flow over time and identifying the points where the flow varied (or changed) significantly. These criteria were then utilized to generate the patient flow categorization bins. We then employed Vilkko, Riitta, et al.'s classification to decide the threshold of the classification of outflow, which is a quiet month (less than 50% of the mean), optimal month (greater than 50% of the mean to less than two times the mean), and busy month (greater than two times the mean or more).

d. Besides accuracy, how did these models perform? Accuracy is not an appropriate measure of model performance on its own - recommend also reporting AUROC, FPR, TPR, etc. Also, consider using a one-versus-rest (OVR) approach to this multi-class problem

In response to the reviewer's comment AUROC, FPR, TPR, and F-score model evaluations are included in the current manuscript. Furthermore, the one-versus-rest (OVR) approach to this multi-class problem experimentation is included in the manuscript.

e. I recommend not balancing the data (see https://doi.org/10.1093/jamia/ocac093 for example), especially if there is interest in interpreting these models. Instead, it is better to choose a different decision threshold and/or use a different performance measure than accuracy (see point d)

Thank you for bringing up an important point about data balance and its potential impact on model interpretation. Oversampling minority classes or undersampling majority classes can indeed lead to biased models that perform well on the training set but fail to generalize to new, unseen data. This is because the model has learned the noise in the training data rather than the underlying patterns. However, we prefer the data imbalance technique for two major purposes: (i) to compare and contrast the performance of the original unbalanced dataset with that of the balanced dataset to observe performance prediction; and (ii) to employ the benefits, including preventing overfitting, promoting diversity in representations, enhancing model interpretability, reducing noise, and fostering fairness. While there are arguments for both approaches, data imbalance can offer benefits like preventing the model from becoming too reliant on the majority class, encouraging the model to focus on the minority class, and helping to reduce bias and discrimination. Furthermore, our study was based on aggregated data. We will take the recommendation into account when we use the raw data, which is useful for model interpretation.

2. Regarding the visualization component of this article, it is not clear what the different Sankey diagrams are supposed to demonstrate. Sankey plots are primarily for visualizing the flow of some quantity, but here they are used also to show **admission rate, length of stay, and bed occupancy rate**. These quantities may be better visualized using other plot types. I also suggest further discussion of the diagrams in the body of the text.

Thank you for bringing up an important point. We appreciate the feedback and have applied the necessary revisions and updates to Figure 4. We added ROC graphs such as Figures 5, 6, 7, and 8 to the updated manuscript. However, we eliminated the length of stay and bed occupancy figures and described using text only to improve the quality of the text. Furthermore, since the manuscript focused on patient flow between the health facilities and not within the health facilities, we feel additional illustrations are not crucial.
