# Supplementary material for: Developing a patient flow visualization and prediction model using aggregated data for a healthcare network cluster in Southwest Ethiopia
Source: PLOS Digit Health. 2023 Nov 8;2(11):e0000376. doi: 10.1371/journal.pdig.0000376 (PMC10631638; doi:10.1371/journal.pdig.0000376)
Supplement: Attachment — Submitted filename: Review_report_plos_one.docx [file pdig.0000376.s002.docx]

Review Report

Reasons given with detailed explanation for research focus is considered valid and fully addresses the problem under consideration. It therefore address concerns raised in the first review.
